# Recurrence of Anti-melanoma Differentiation-Associated Gene 5 Antibody-Positive Dermatomyositis Following Long-Term Remission: A Report of Two Cases and Review of Literature

**DOI:** 10.7759/cureus.88849

**Published:** 2025-07-27

**Authors:** Hironobu Komine, Takao Nagashima, Hiroki Yabe

**Affiliations:** 1 Division of Rheumatology, Department of Medicine, Jichi Medical University Saitama Medical Center, Saitama, JPN; 2 Division of Rheumatology and Clinical Immunology, Department of Medicine, Jichi Medical University, Shimotsuke, JPN

**Keywords:** cutaneous ulcer, cyclophosphamide, interstitial lung disease, liver dysfunction, polymyositis

## Abstract

We report two cases of anti-melanoma differentiation-associated gene 5 (MDA5) antibody-positive dermatomyositis (DM) that recurred after long-term remission. The first case involved a 41-year-old Japanese woman, and the other involved a 31-year-old Chinese woman. Both patients were initially treated with prednisolone, tacrolimus, and low-dose intravenous cyclophosphamide. Antibody negativity was confirmed during remission. However, DM recurred in both patients after 8 and 10 years, respectively, accompanied by the reappearance of anti-MDA5 antibodies. The Japanese patient exhibited severe liver dysfunction at both onset and recurrence. In contrast, the Chinese patient presented with clinically amyopathic DM and had mild respiratory and cutaneous symptoms at both time points. The recurrence patterns were similar in both cases. A review of recurrent anti-MDA5 antibody-positive DM cases revealed common features, including female predominance, mild interstitial lung disease, low serum ferritin levels, and relatively low cumulative cyclophosphamide doses.

## Introduction

Dermatomyositis (DM) is an idiopathic inflammatory myopathy characterized by proximal muscle weakness and distinctive skin manifestations such as Gottron’s sign and heliotrope rash. Common comorbidities associated with DM include interstitial lung disease (ILD) and malignancies [1]. Disease-specific autoantibodies are frequently detected in the sera of patients with DM, including anti-transcription intermediary factor 1-γ, anti-nuclear matrix protein 2, anti-Mi-2, anti-small ubiquitin-like modifier-activating enzyme, and anti-melanoma differentiation-associated gene 5 (MDA5) antibodies. Each of these autoantibodies is associated with a distinct clinical phenotype in terms of cutaneous findings, systemic involvement, and malignancy risk [1].

The anti-MDA5 antibody is particularly common in Asian patients, with a reported prevalence ranging from 11% to 60% [2]. Patients with anti-MDA5 antibodies typically present with unique clinical and laboratory features, including palmar papules, cutaneous ulcers, arthralgia, minimal muscle weakness, liver dysfunction, low serum creatine kinase (CK) levels, elevated serum ferritin levels, and ILD [2,3]. Biomarkers such as serum ferritin, lactate dehydrogenase (LDH), and Krebs von den Lungen-6 (KL-6) are useful in evaluating ILD activity, with elevated levels often indicating a poor prognosis [3]. ILD in these patients can progress rapidly, with reported mortality rates as high as 50%, although recent studies suggest this has decreased to approximately 23%-26% [2,4,5]. Conversely, patients who survive beyond six months typically have a favorable long-term prognosis, and relapse is considered rare [4,6].

Although uncommon, several case reports have documented recurrence after prolonged remission in patients with anti-MDA5 antibody-positive DM [7-11]. However, the clinical and laboratory characteristics of patients who experience recurrence remain unclear. Here, we report two such cases with recurrence occurring 8 and 10 years after initial disease onset, respectively, and review the literature to help clarify the features associated with recurrence in anti-MDA5 antibody-positive DM.

## Case presentation

Case 1

**Figure 1:**
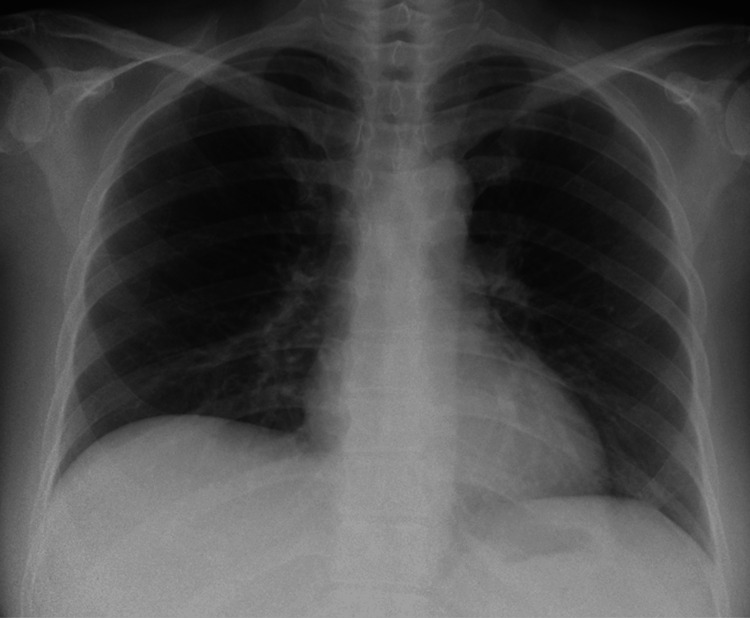
Chest radiograph of the first case No significant findings were evident.

**Figure 2:**
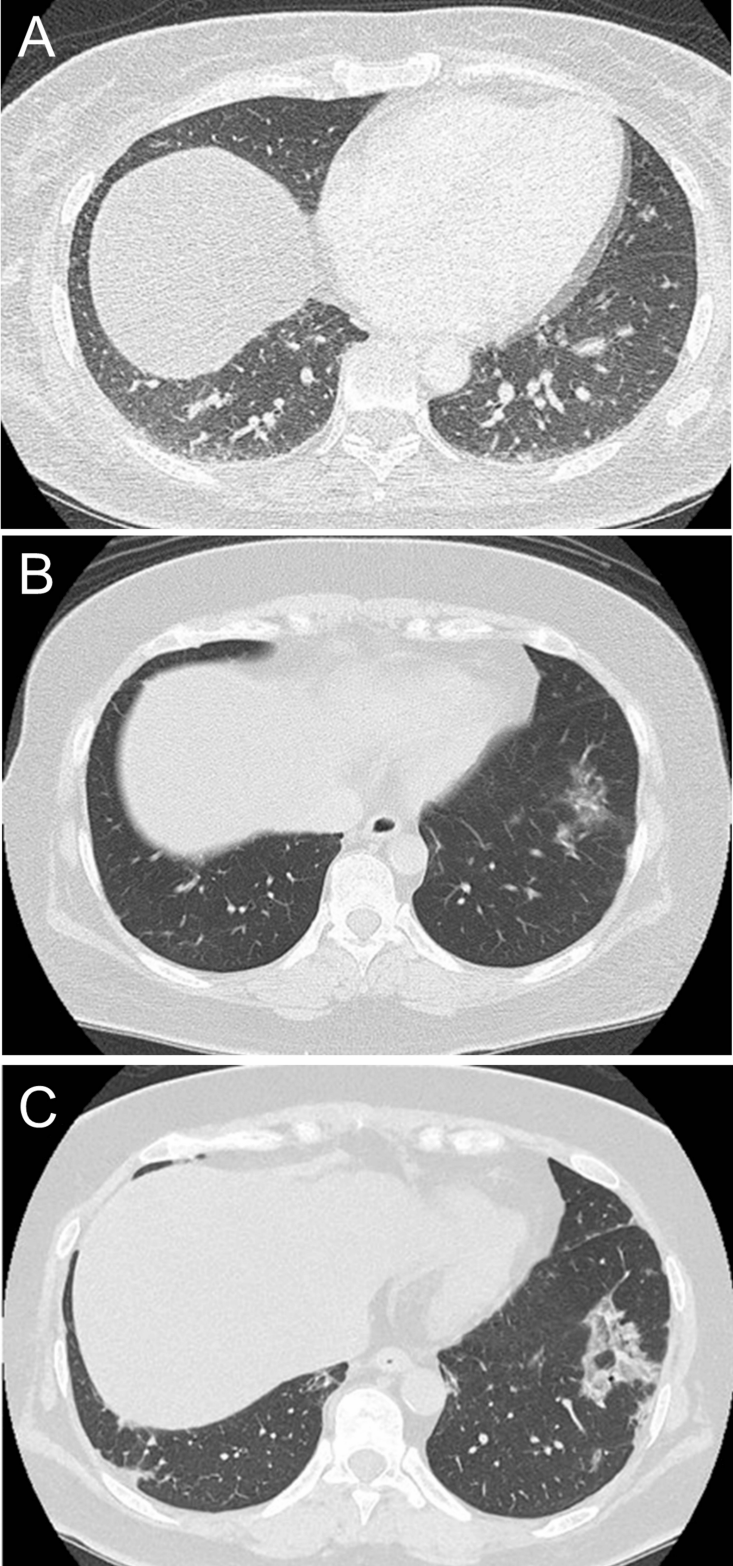
Chest computed tomography of the first case (A) Ground-glass opacities at the time of diagnosis in bilateral dorsal lung fields. (B) Ground-glass opacity only in the base of the left lung at the first recurrence. (C) Development of a new consolidation in the lower part of the left lung at the second recurrence.

A 41-year-old Japanese woman with no significant medical history was admitted to our hospital with persistent fever, erythema on the face and upper arms, arthralgia, and muscle weakness, which had gradually progressed over eight months. Physical examination revealed heliotrope rash, Gottron’s sign on the dorsum of both hands, and proximal muscle weakness. Fine crackles were heard on chest auscultation. The laboratory findings were as follows (Table 1): aspartate aminotransferase (AST) level, 552 U/L (reference, <31 U/L); alanine aminotransferase (ALT) level, 275 U/L (reference, <31 U/L); CK level, 49 U/L (reference, <143 U/L); and KL-6 level, 496 U/mL (reference, <435 U/mL). The anti-MDA5 antibody titer was >150 index (reference, <32) (MESACUP^TM^ anti-MDA5 antibody test, MBL, Tokyo, Japan), but no respiratory distress was evident. Oxygen saturation was 98% on ambient air, but the respiratory function test results revealed a mildly decreased forced vital capacity of 80.2%. The chest radiograph showed no significant findings (Figure 1), but chest computed tomography (CT) revealed mild ground-glass opacities in both dorsal lung fields (Figure 2A). Magnetic resonance imaging (MRI) revealed a high-intensity area in the gluteus muscle on short tau inversion recovery images. The patient was diagnosed with DM based on the European League Against Rheumatism/American College of Rheumatology criteria [12], which was treated with a combination therapy of 50 mg/day prednisolone (0.8 mg/kg), 4 mg/day tacrolimus, and 500 mg intravenous cyclophosphamide (IVCY) administered four times biweekly (2 g in total). Thereafter, muscle weakness, liver function test results, and skin rashes gradually improved. Liver function test results returned to normal, and the patient was discharged two months after treatment. Skin ulcers persisted, requiring several months for complete healing.

**Table 1:** Laboratory findings on admission of the first case MDA5: melanoma differentiation-associated gene 5.

Blood analyses	Result	Reference range
Hemoglobin (g/dL)	11.7	11.3-15.2
Leukocyte (×10^9^/L)	7.37	3.5-9.1
Platelet count (×10^9^/L)	481	130-369
Blood urea nitrogen (mg/dL)	10	8-20
Creatinine (mg/dL)	0.28	0.46-0.79
Aspartate aminotransferase (U/L)	552	13-30
Alanine aminotransferase (U/L)	275	7-23
Lactate dehydrogenase (U/L)	566	124-222
Alkaline phosphatase (U/L)	899	106-322
Gamma-glutamyl transferase (U/L)	717	9-32
Creatine kinase (U/L)	49	41-153
Aldolase (U/L)	24.5	2.1-6.1
C-reactive protein (mg/dL)	0.89	<0.14
IgG (mg/dL)	2,715	870-1700
Krebs von den Lungen-6 (U/mL)	496	105-435
Serum ferritin (ng/mL)	107.8	4.2-136.7
Anti-MDA5 antibody (index)	≥150	<32

The patient experienced relapse 1.5 years later, accompanied by skin rash, cutaneous ulcers, and worsening of ILD while on 6 mg/day prednisolone and 4 mg/day tacrolimus. Laboratory tests revealed elevated C-reactive protein (CRP; 8.11 mg/dL) and KL-6 (457 U/mL) levels, but normal leucocyte count (6,980/μL), CK (41 U/L), and serum ferritin levels (88.1 ng/mL). Chest CT revealed mild worsening of ILD (Figure 2B). The anti-MDA5 antibody titer remained positive at >150 index. Prednisolone dose was increased to 40 mg/day, and 500 mg IVCY was administered once. Two years after the relapse, the anti-MDA5 antibody test result was negative for the first time. This negative result continued for >3 years; therefore, the doses of both prednisolone and tacrolimus were gradually decreased and discontinued seven years after disease onset. As the disease was considered cured, outpatient follow-up was discontinued.

**Figure 3:**
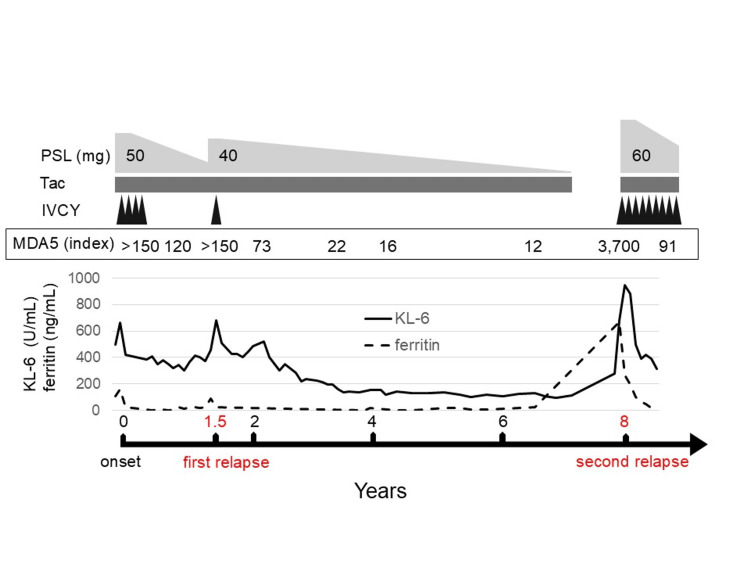
Clinical course of the first case KL-6: Krebs von den Lungen-6, MDA5: anti-melanoma differentiation-associated gene 5 antibody, PSL: prednisolone, Tac: tacrolimus, IVCY: intravenous cyclophosphamide.

One year later, the patient was readmitted with fever, shortness of breath, and generalized muscle weakness. Erythema was observed on the upper limbs, hands, chest, abdomen, and back. Muscle strength was determined as 4/5 using manual muscle testing in the proximal muscles. Laboratory tests revealed elevated AST (487 U/L), ALT (179 U/L), CK (350 U/L), KL-6 (678 U/mL), serum CRP (1.23 mg/dL), and ferritin (668.9 ng/mL) levels and reappearance of the anti-MDA5 antibody (3,700 index). Oxygen saturation was 98% on ambient air, but the respiratory function test result revealed a forced vital capacity of 84.1%. Chest CT revealed patchy consolidations in bilateral lung fields (Figure 2C), leading to the diagnosis of recurrent DM. The disease severity was comparable between the initial presentation and disease recurrence for this patient. Combination treatment with 60 mg/day prednisolone (0.8 mg/kg), 4 mg/day tacrolimus, and 1200 mg IVCY was initiated. Subsequently, muscle weakness gradually improved. IVCY was continued with tapering doses of prednisolone. Although respiratory symptoms, muscle weakness, and liver abnormalities resolved, recalcitrant cutaneous ulcers developed on the dorsal and palmar surfaces of the hands and persisted for eight months, despite low serum ferritin levels (Figure 3).

Case 2

**Figure 4:**
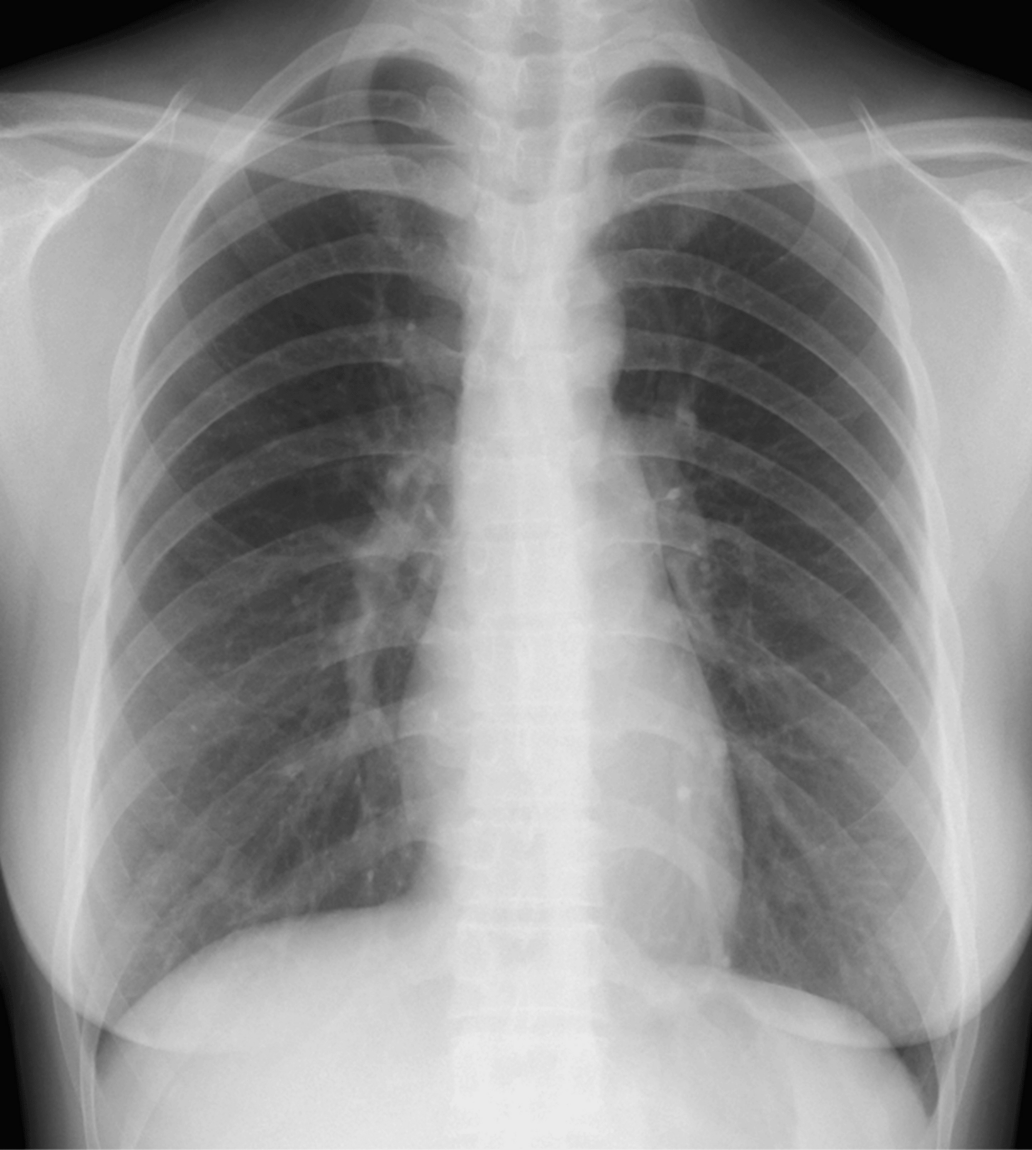
Chest radiograph of the second case No significant findings were evident.

**Figure 5:**
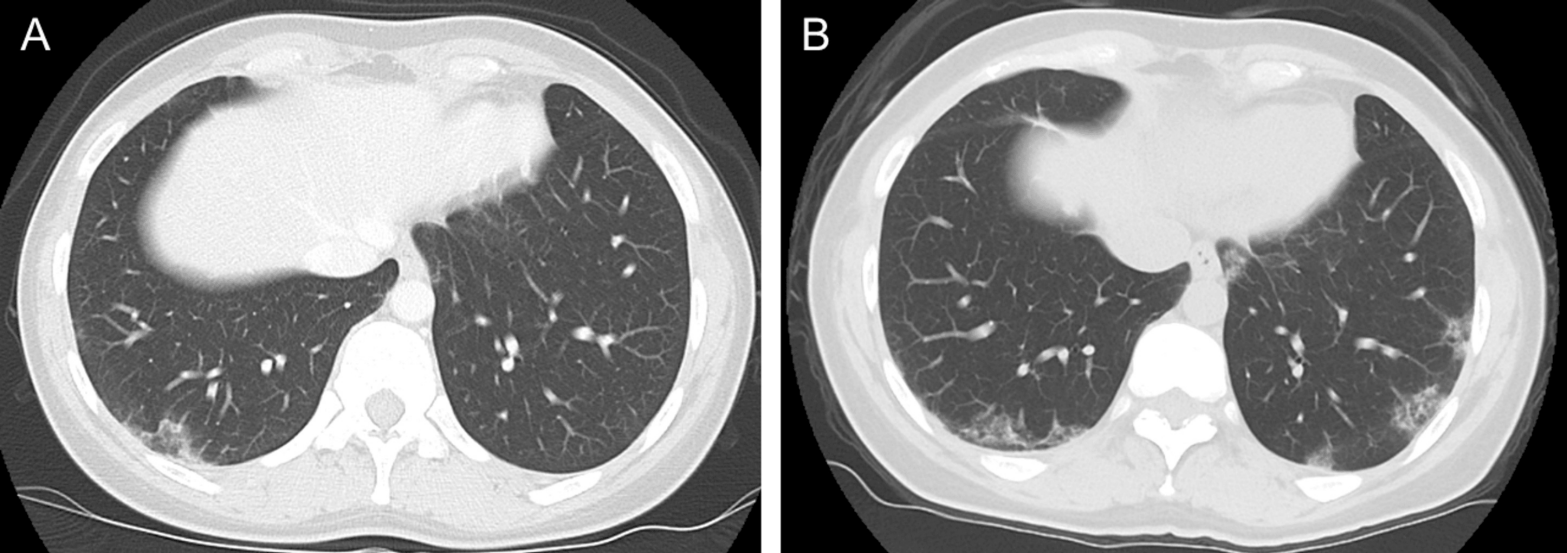
Chest computed tomography of the second case (A) Ground-glass opacity only in the base of the right lung. (B) Multiple ground-glass opacities in bilateral lung bases on recurrence.

A 31-year-old Chinese woman with no significant medical history was diagnosed with clinically amyopathic DM based on typical cutaneous symptoms, including heliotrope rash, Gottron’s sign, shawl sign, and mechanic’s hands. Low-grade fever, arthralgia, and swollen fingers were present, without any respiratory symptoms or weakness. Laboratory findings were as follows (Table 2): AST level, 43 U/L; ALT level, 33 U/L; CK level, 169 U/L; aldolase level, 9.0 U/L; CRP level, 0.09 mg/dL; serum ferritin level, 209.6 ng/mL; KL-6 level, 309 U/mL; and anti-MDA5 antibody titer, 193 index (MESACUP^TM^ anti-MDA5 antibody test). Oxygen saturation was 100%, and the respiratory function test result revealed a forced vital capacity of 116.6%. Chest radiograph showed no significant findings (Figure 4). Chest CT revealed mild ground-glass opacities in the right lung (Figure 5A). MRI scan of the thighs revealed no abnormal intensities. The patient was treated with 50 mg/day prednisolone (1 mg/kg), 4 mg/day tacrolimus, and biweekly 500 mg IVCY. Two years later, the anti-MDA5 antibody test result became negative (13 index). Prednisolone was discontinued six years later, and only tacrolimus was continued (<1 mg/day). As there were no subjective symptoms, the patient may have believed that the disease was cured, independently discontinued outpatient visits, and ceased taking the prescribed medication 8.5 years later. 

**Table 2:** Laboratory findings on admission of the second case MDA5: melanoma differentiation-associated gene 5.

Blood analyses	Result	Reference range
Hemoglobin (g/dL)	12.7	11.3-15.2
Leukocyte (×10^9^/L)	4,400	3.5-9.1
Platelet count (×10^9^/L)	134	130-369
Blood urea nitrogen (mg/dL)	11	8-20
Creatinine (mg/dL)	0.47	0.46-0.79
Aspartate aminotransferase (U/L)	43	13-30
Alanine aminotransferase (U/L)	33	7-23
Lactate dehydrogenase (U/L)	352	124-222
Alkaline phosphatase (U/L)	221	106-322
Gamma-glutamyl transferase (U/L)	62	9-32
Creatine kinase (U/L)	169	41-153
Aldolase (U/L)	9.0	2.1-6.1
C-reactive protein (mg/dL)	0.09	<0.14
IgG (mg/dL)	1,379	870-1700
Krebs von den Lungen-6 (U/mL)	309	105-435
Serum ferritin (ng/mL)	209.6	4.2-136.7
Anti-MDA5 antibody (index)	193	<32

Ten years after the onset, the patient was readmitted because of a relapse of arthralgia, skin rash, and shortness of breath. Laboratory findings revealed normal levels of CK (135 U/L), KL-6 (431 U/mL), and serum ferritin (57.9 ng/mL). Anti-MDA5 antibody titers were elevated at 600 index. Anti-Mi-2 antibody test result was negative (MESACUP^TM^ anti-Mi-2 test). Oxygen saturation was 97%, and the respiratory function test result revealed a forced vital capacity of 113.3%. Chest radiograph revealed no relevant findings. CT revealed scattered ground-glass opacities in both lungs (Figure 5B). The patient was treated with 50 mg/day prednisolone (0.9 mg/kg), 3 mg/day tacrolimus, and 1000 mg IVCY, with a good response.

## Discussion

We herein report two cases of DM positive for the anti-MDA5 antibody that recurred after long-term remission. Recurrence occurred 8 and 10 years after the onset, respectively. The anti-MDA5 antibody reappeared in both patients at the time of recurrence. The higher antibody titers at recurrence in both cases do not indicate a dramatic increase in the antibody levels during the episode; this can be attributed to the change in the antibody detection method by the company. Both patients showed a good treatment response for ILD; however, the cutaneous ulcers in Case 1 were resistant to treatment.

The anti-MDA5 antibody was present at recurrence in both cases. It is not only a marker of distinct DM subtypes, but its reappearance also indicates recurrence [9,11]. Recurrence occurred in Case 1 despite more than three years of persistent antibody negativity. In Case 2, the persistence of the antibody negativity was not confirmed, but it may have continued for >6 years. Anti-MDA5 antibody-positive DM can recur even after >3 years of antibody negativity [11]. Although the prognosis is favorable in patients who survive for more than 6-12 months, the risk of recurrence persists as long as antibody positivity persists [11,13,14]. Whether serial examination of serum KL-6 level can detect recurrence of ILD before that of anti-MDA5 antibody level is not yet elucidated.

The recurrence rate of anti-MDA5 antibody-positive DM ranges from 10% to 68%, depending on the treatment, race, and follow-up period [4,5,11,13]. The lowest recurrence rate has been reported in patients treated with a triple combination therapy consisting of glucocorticoids, tacrolimus, and sufficient doses of IVCY (median of approximately 9 g) [4]. Conversely, in a study showing the highest recurrence rate, cyclophosphamide was not used (only glucocorticoids and calcineurin inhibitors), and multiple recurrences occurred (37.5%) [13]. The triple combination therapy has shown a lower recurrence rate than mono- or dual therapy (glucocorticoids and calcineurin inhibitors or IVCY) [4]. In another study reporting a five-year recurrence rate of 22%, three-quarters of the patients received cyclophosphamide (dose not specified) [5]. These results indicate that administration of cyclophosphamide in sufficient doses may be necessary to prevent recurrence.

Recurrence cannot be predicted based on baseline clinical characteristics. One study showed that baseline serum ferritin and LDH levels were higher in patients who experienced recurrence than in those who attained long-term remission [13]. In that study, cyclophosphamide was not used in the treatment regimen, and anti-MDA5 antibodies persisted in approximately half of the patients at the end of follow-up. As cyclophosphamide was not administered, persistent antibody positivity may have affected the risk of recurrence. In other studies, baseline characteristics, including serum ferritin, CRP, and LDH levels, were not significant risk factors for recurrence [4,5]. Furthermore, the discontinuation of immunosuppressive agents owing to adverse events may also affect recurrence [4].

Cutaneous ulcers persisted in Case 1 even after improvement in muscle and respiratory symptoms. Cutaneous ulcers were also evident in a few cases of recurrence [9,10]. Similar to that in our patient, long-term treatment with tofacitinib was necessary to achieve a cure (six months) [10]. Cutaneous ulcers have been suggested as a poor prognostic factor for ILD [15], but a study has shown conflicting results [16]. Managing cutaneous ulcers is challenging, but it has received less attention than ILD, probably because it does not directly impact mortality. Several treatments have been attempted, including intravenous immunoglobulin, mycophenolate mofetil, vasodilators including endothelin receptor inhibitors, and phosphodiesterase inhibitors [17].

We reviewed cases of recurrent DM that tested positive for anti-MDA5 antibodies (Table 3) [7-11]. We excluded patients who were persistently positive for antibodies because they were considered to have received insufficient induction treatment [14]. All reported cases were from Japan, and all patients, except one, were Japanese women. Not all patients were confirmed to have negative anti-MDA5 antibody test results. Some patients who experienced early recurrence (within two years) may have had persistent anti-MDA5 antibodies, as observed in Case 1 [8,10]. In our review (n = 9), all recurrences occurred in female patients, and 78% of the patients did not have RP-ILD. No cyclophosphamide was administered in three, a dose of 3 g or less was administered in four, and the total dose was not specified for two patients. Serum ferritin and KL-6 levels were low in patients positive for anti-MDA5 antibody. The reason for low doses of cyclophosphamide administration in such cases may be a favorable treatment response or the unavailability of anti-MDA5 antibody detection systems. Although the female-to-male prevalence ratio is approximately 2-3:1 in anti-MDA5 antibody-positive DM [4,5,11], recurrences occur exclusively in females. Since most recurrences occurred in Japanese patients, it is unclear whether these features are common in other ethnicities, and further research is needed.

**Table 3:** Cases of recurrence of anti-MDA5 antibody-positive DM AZA: azathioprine, BMZ: betamethasone, CY: cyclophosphamide, CyA: cyclosporine, DM: dermatomyositis, F: female, ILD: interstitial lung disease, IP: immunoprecipitation assay, IVCY: intravenous cyclophosphamide, IVIg: intravenous immunoglobulin, IVMP: intravenous methylprednisolone pulse, KL: Krebs von den Lungen, MDA5: melanoma differentiation-associated gene 5, MMF: mycophenolate mofetil, ND: not described, PMX: polymyxin B-immobilized fiber column direct hemoperfusion, PH: pulmonary hypertension, PSL: prednisolone, RP-ILD: rapidly progressive-interstitial lung disease, Tac: tacrolimus, TOF: tofacitinib.

Study	Age/sex	Recurrence (years after)	RP-ILD	Treatment	Total CY dose (g)	Serum KL-6 level (U/mL)	Serum ferritin level (ng/mL)	Anti-MDA5 Ab titer (index)	Prognosis
Matsushita et al. (2017) [11]	78/F	-	Yes	PSL, Tac, IVCY	0.5 g/m^2 ^× 2 times (dose ND)	ND	1,500	250->neg	-
	-	5	-	PSL, Tac, IVCY, IVIg	0.5 g/m^2 ^× 4 times (dose ND)	ND	600	200	Survived
Sato et al. (2017) [8]	59/F	-	No	PSL	0	1,147	ND	ND	-
	-	1	-	PSL, CyA (discontinued)	0	900	ND	ND	-
	-	4	-	IVMP, PSL	0	500	ND	ND	-
	-	9	-	IVMP, PSL, AZA, Tac	0	652	162	Positive (IP)	Survived
Endo et al. (2018) [7]	70/F	-	No	IVMP, PSL, CyA, IVCY	5 times (dose ND)	ND	ND	148->ND	-
	-	7	-	IVMP, PSL, CyA, IVCY	2	274	319	109	Survived
Ishikawa et al. (2020) [10]	57/F	-	No	PSL, Tac, IVCY	3	ND	ND	Positive->ND (titer ND)	-
	-	1.8	-	PSL, TOF	0	1,271	197.8	>150	Survived
Suzuki et al. (2021) [9]	44/F	-	Yes	IVMP, PSL, CyA, IVCY	2	603	47.1	ND	-
	-	3.5	-	IVMP, BMZ, Tac, IVCY, IVIg, PMX	3	1,370	19.3	ND	-
	-	7.8	-	PSL, Tac, IVCY	3	1,160	13.2	Neg->116	Survived
Suzuki et al. (2021) [9]	54/F	-	No	IVMP, PSL, CyA	0	521	ND	ND	-
	-	7	-	PSL, Tac, IVCY	3	1,480	ND	ND	-
	-	12	-	IVMP, PSL, MMF, Tac, IVCY	0.5	1,270	246.9	196	Dead (PH with ILD)
Suzuki et al. (2021) [9]	60/F	-	No	IVMP, PSL, CyA	0	537	592.9	ND	-
	-	7	-	BMZ, Tac, IVCY	3	785	303	170	Survived
Case 1	41/F	-	No	PSL, Tac, IVCY	2	496	107.8	>150	-
	-	1.5	-	PSL, Tac, IVCY	0.5	457	88.1	>150->neg	-
	-	8	-	PSL, Tac, IVCY	7.4	678	668.9	3,700	Survived
Case 2	31/F	-	No	PSL, Tac, IVCY	2	309	209.6	193->neg	-
	-	10	-	PSL, Tac, IVCY	3.7	431	57.9	600	Survived

## Conclusions

We report two cases of DM in which recurrence was accompanied by the reappearance of anti-MDA5 antibodies. These antibodies may reemerge even after long-term remission, highlighting the importance of extended follow-up, even when antibody tests become negative. Early detection of recurring anti-MDA5 antibodies allows timely intervention before the onset of interstitial lung disease or skin lesions, helping prevent further disease progression.
